# Multitarget Activities of Kleeb Bua Daeng, a Thai Traditional Herbal Formula, Against Alzheimer’s Disease

**DOI:** 10.3390/ph13050079

**Published:** 2020-04-25

**Authors:** Chantha Chheng, Pornthip Waiwut, Kusawadee Plekratoke, Yaowared Chulikhit, Supawadee Daodee, Orawan Monthakantirat, Supaporn Pitiporn, Natdanai Musigavong, Pakakrong Kwankhao, Chantana Boonyarat

**Affiliations:** 1 Faculty of Pharmaceutical Sciences, Khon Kaen University, Khon Kaen 40002, Thailand; chheng_chantha72@yahoo.com (C.C.); kusawadee2535@gmail.com (K.P.); yaosum@kku.ac.th (Y.C.); csupawad@kku.ac.th (S.D.); oramon@kku.ac.th (O.M.); 2 Faculty of Pharmaceutical Sciences, Ubon Ratchathani University, Ubon Ratchathani 34190, Thailand; pwaiwut79@yahoo.com; 3 Chao Phya Abhaibhubejhr Hospital, Prachin Buri 25000, Thailand; spitiporn@yahoo.com (S.P.); mnatdanai@hotmail.com (N.M.); pakakrong2@gmail.com (P.K.)

**Keywords:** antioxidant, acetylcholinesterase inhibition, beta-amyloid aggregation, neuroprotection, *Nelumbo nucifera*, *Piper nigrum*, *Centella asiatica*

## Abstract

The Kleeb Bua Daeng formula (KBD) is a Thai traditional medicine for brain health promotion. On the basis of the activities of its individual components, the KBD could have good potential for the treatment of Alzheimer’s disease (AD). Herein, we investigated the KBD as an AD treatment. The ethanol extracts of KBD and its components, i.e., *Nelumbo nucifera* (NN), *Piper nigrum* fruits (BP), and the aerial part of *Centella asiatica* (CA) exhibited antioxidant activity, as determined by both ABTS and DPPH assays. The Ellman’s assay revealed that the KBD, NN, and BP showed an ability to inhibit acetylcholinesterase. The thioflavin T assay indicated that the KBD, NN, BP, and CA inhibited beta-amyloid aggregation. The neuroprotection and Western blot analysis revealed that the KBD reduced H_2_O_2_-induced neuronal cell death by inhibiting the expression of pro-apoptotic factors, i.e., cleaved caspase-9 and -3, p-P65, p-JNK, and p-GSK-3β, as well as by inducing expression of anti-apoptotic factors, i.e., MCl_1_, BCl_xl_, and survivin. Furthermore, the KBD could improve scopolamine induced memory deficit in mice. Our results illustrate that the KBD with multimode action has the potential to be employed in AD treatment. Thus, the KBD could be used as an alternative novel choice for the prevention and treatment of patients with AD.

## 1. Introduction

Alzheimer’s disease (AD) is a multifactorial neurological disorder categorized by memory impairment, progressive decline in cognitive functions, and changes in behaviors and moods [1]. The incidence rate of AD has presented an exponential increase, particularly in people over 65 years of age; the disease currently affects over 33 million people worldwide [2,3,4]. Thus, AD is essentially a troublesome disease for the public health systems of the world.

The pathogenesis of AD follows numerous pathways which include deficiency of acetylcholine neurotransmitters [5], extracellular accumulation of beta amyloid plaques [6], intracellular deposit of neurofibrillary tangles [7,8] and oxidative stress leading to neuronal cell death [9,10]. Over the past decades, many pharmacological strategies have been devoted to target these pathways, including both cholinergic and noncholinergic interventions [11,12]. The current therapeutic approaches for AD include the following: (a) acetylcholinesterase (AChE) inhibitors such as donepezil, rivastigmine, and galantamine and (b) the NMDA receptor antagonist, memantine [13]. However, while these drugs can be used for reducing symptoms, they are not effective for curing AD. Therefore, an effective pharmacotherapy against AD is urgently needed.

Due to the multiple pathways involved in the AD pathogenesis, the classical single-target approach that modulates one target is insufficient. Therefore, searching for candidates that target multiple stages of the pathogenesis cascade has become an effective strategy for designing pharmacotherapy against AD [14,15,16]. Thus, natural products composed of diverse phytochemical constituents, with multi-action or synergistic effects targeting multiple sites of the pathogenic cascade of AD, could afford additional benefits for AD therapy [17,18,19].

The Kleeb Bua Daeng formula (KBD) has been used for a long time by local healers in Thailand as a multi-herb folk medicine for the treatment of patients who have declining memory function and insomnia. Since June 2013, the KBD has been prescribed by doctors in the Chao Phya Abhaibhubejhr Hospital, Thailand as a traditional medicine for brain tonic, sleep aid, mood stabilizer, and relaxation [20]. The KBD consists of the following three components: (i) *Nelumbo nucifera* petal (NN), (ii) *Piper nigrum* fruit (BP), and (iii) the aerial part of *Centella asiatica* (CA) mixed in a ratio of 1:1:1 (dry weight). Interestingly, each component of the KBD has been reported to have biological activity relevant to AD. *Nelumbo nucifera* has been extensively used as a component of traditional Thai, Chinese, Indian, Japanese, and Korean medicines [21]. Several investigations have reported that different parts of NN have activities related to AD patients’ cognitive functions, including inhibition of AChE and β-site APP cleaving enzyme 1 (BACE1) [22]. In addition, NN reversed scopolamine-induced cognitive impairment in mice by increasing choline acetyltranferase (ChAT) expression [23]. *Piper nigrum*, or black pepper, has been widely utilized in several traditional medicine systems such as the Unani and Ayurvedic systems [24]. Black pepper has been reported as a potential treatment for cognitive dysfunction and neurodegeneration in AD [25,26,27]. *Centella asiatica* has been used for a long time in Ayurvedic traditions as a brain tonic and a memory enhancer [28]. There is evidence to show that, in animal models, CA could enhance memory retention and improve learning performance. The mechanisms for enhancing the cognitive function of CA have included the reduction of ß-amyloid aggregation and protection of brain damage [29,30]. On the basis of the activities of its components, the KBD could have good potential as a novel treatment for AD. However, there has been no previous study investigating the KBD as a therapeutic for AD. 

This current study has investigated the effects of the KBD and its components on the following four targets in the pathological cascade of AD: (i) antioxidant activity, (ii) cholinesterase function, (iii) beta amyloid aggregation, and (iv) neuroprotection *in vitro*. The expression levels of proteins involved in neuronal cell death were determined in order to understand the mechanism of neuroprotection. Finally, the effects of the KDB on cognitive function were investigated in a scopolamine-induced cognitive-deficit mouse model.

## 2. Results

### 2.1. Total Phenolic and Total Flavonoid Contents 

Phenols and flavonoids from plants can demonstrate several pharmacological activities. In order to study the relationship between activities and phytochemicals, the total phenolic and total flavonoid contents in ethanolic extracts of the KBD and its constituents were determined, and the results are shown in Table 1.

### 2.2. Antioxidant Activities

**Figure 1:**
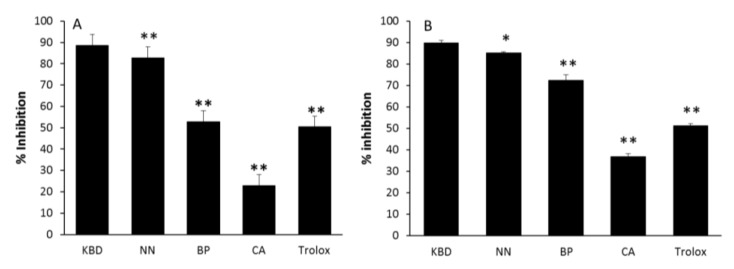
The effect of ethanol extracts of the KBD and its components on ABTS (**A**) and DPPH (**B**) radical scavenging action. The KBD extract of 3 mg/mL (KBD) and its components at equivalent amounts, i.e., *Nelumbo nucifera* 1 mg/mL, (NN); *Piper nigrum* 1 mg/mL (BP), and *Centella asiatica* 1 mg/mL (CA), were evaluated for radical scavenging action by ABTS and DPPH assay. The values are reported as means ± SD (*n* = 5). * *p* < 0.05 and ** *p* < 0.01 as compared with the KBD group. Trolox at the concentration of 80 and 20 μM was used as a positive control in ABTS and DPPH assay, respectively.

The alleviation of oxidative stress is a crucial strategy in designing agents for AD treatment. The antioxidant activities of the ethanol extracts of the KBD and its constituents were examined by using the ABTS and DPPH radical scavenging assays. The ability of extracts to scavenge radicals is shown as IC_50_, the test compound concentration that resulted in 50% inhibition of free radicals. The KBD extract scavenged both ABTS and DPPH radicals with IC_50_ values of 0.90 ± 0.06 and 0.62 ± 0.06 mg/mL, respectively, as shown in Table 2. Trolox, used as a reference standard, could scavenge ABTS and DPPH radicals with IC_50_ of 73.14 ± 2.71 and 22.91 ± 0.16 µM, respectively. To compare the activities among the components, the KBD extract at 3 mg/mL and its components at equivalent amounts (NN, BP, and CA at 1 mg/mL) were assayed. The NN extract possessed the most potent radical scavenging activity, followed by BP and CA (Figure 1). Our results showed that the radical scavenging activity of the KBD is mainly provided by NN.

### 2.3. In Vitro Study of AChE Inhibitory Activity

**Figure 2:**
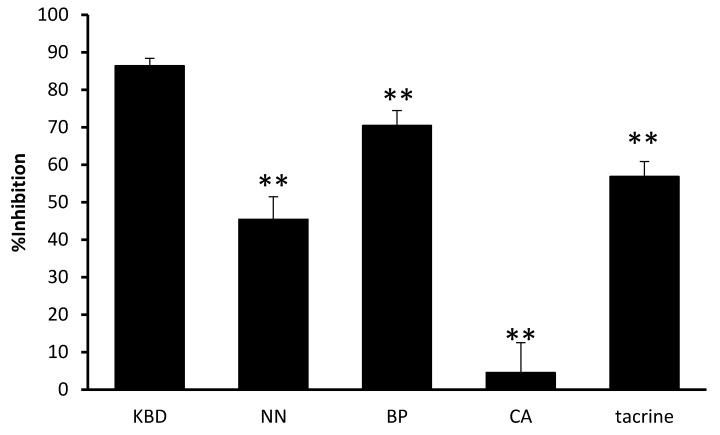
The effect of the KBD and its components on AChE activity. The KBD extract 3 mg/mL (KBD) and its components at equivalent amount, i.e., *Nelumbo nucifera* 1 mg/mL, (NN), *Piper nigrum* 1 mg/mL (BP), and *Centella asiatica* 1 mg/mL (CA) were evaluated for AChE inhibitory action by Ellman’s assay. Data are reported as means ± SD (*n* = 3). ** *p* < 0.01 as compared with the KBD group. Tacrine 0.30 µM was used as a positive control.

The KBD and its constituents were evaluated for their anti-acetylcholinesterase activity. The results are represented by IC_50_ values (Table 2). The KBD extract inhibited AChE with an IC_50_ value of 2.17 ± 0.23 mg/mL, whereas its components NN and BP inhibited 50% of AChE function at concentrations of 1.88 ± 0.10 and 0.93 ± 0.12 mg/mL, respectively. CA showed no effect on AChE function at 5 mg/mL. Tacrine, a reference standard, demonstrated AChE inhibitory activity with a IC_50_ value of 0.29 ± 0.03 μM. By comparison among the components, the ethanol extract of BP displayed the most potent AChE inhibitory activity (Figure 2). The KBD at a concentration of 3 mg/mL inhibited AChE function (86.36 percentage inhibitory action), while its components NN, BP, and CA at 1 mg/mL showed varying abilities to inhibit AChE function with percentage inhibitory actions of 45.45%, 70.45%, and 4.55%, respectively. Our results suggest that BP and NN are the components mainly responsible for the anti-acetylcholinesterase activity of the KBD. 

### 2.4. In Vitro Assay for Aβ Aggregation Inhibition

**Figure 3:**
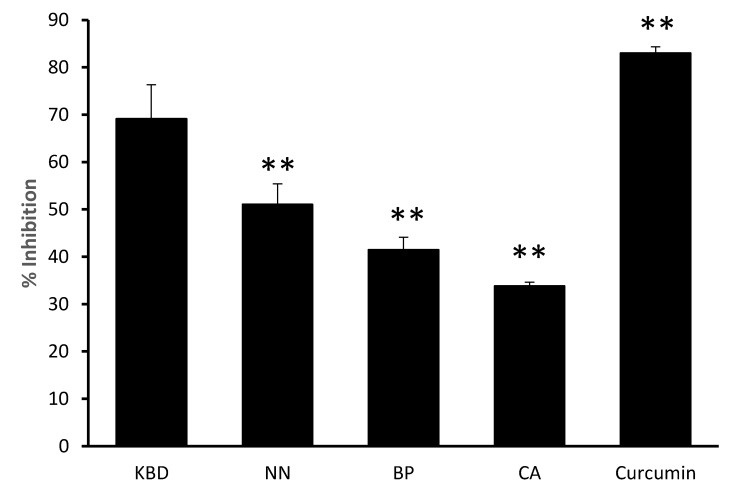
The effect of the KBD and its components on Aβ aggregation. The KBD extract 300 µg/mL (KBD) and its components at equivalent amounts, i.e., *Nelumbo nucifera* 100 µg/mL (NN), *Piper nigrum* 100 µg/mL (BP), and *Centella asiatica* 100 µg/mL (CA) were evaluated for inhibition of Aβ_1-42_ aggregation by ThT assay. Curcumin (10 μM) was used as a positive control. The values are reported as means ± SD (*n* = 3). ** *P* < 0.01 as compared with the KBD group.

The thioflavin T assay revealed inhibition of amyloid β aggregation by the KBD and its components, as shown in Figure 3. Curcumin (10 μM) was used as a positive control. The KBD (300 µg/mL) and its components (100 µg/mL) inhibited Aβ_1-42_ aggregation with percentage inhibitory values ranging from 33.82–9.16%. 

### 2.5. Effect on H_2_O_2_ -Induced Cell Damage in Neuroblastoma Cells

**Figure 4:**
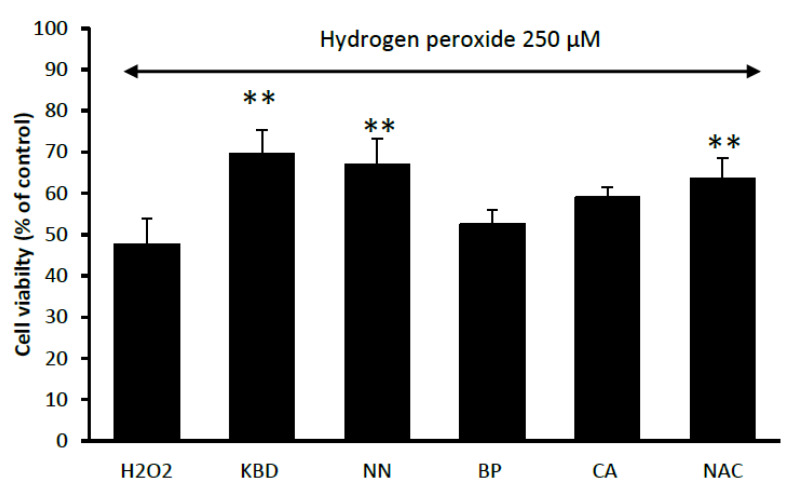
Effect of the KBD 100 μg/mL and its component extracts (*Nelumbo nucifera* (NN), *Piper nigrum* (BP), and *Centella asiatica* (CA) at 35 μg/mL on H_2_O_2_-induced oxidative cell damage in neuroblastoma cells*. N*-acetyl cysteine (NAC) at 100 µg/mL was used as a reference standard. Data are means ± SD (*n* = 5) ** *p* < 0.01 as compared with the H_2_O_2_-treated control group.

The neuroprotective effect of the KBD and its components (NN, BP, and CA) against oxidative stress was investigated by MTT assay in neuroblastoma cell line SH-SY5Y. To induce oxidative damage, hydrogen peroxide (H_2_O_2_) was used and *N*-acetyl cysteine (NAC) at 100 µg/mL was employed as a reference compound. Treatment of the neuronal cells with the KBD extract, at the concentration of 100 μg/mL, significantly increased cell viability as compared with the H_2_O_2_ only control (Figure 4). For its components, tested at the equivalent amount (35 μg/mL), only NN showed a significant increase in cell viability as compared with the H_2_O_2_ only treated control (Figure 4).

### 2.6. The Effect on the Expression of Proteins Related to Neuronal Cell Death

**Figure 5:**
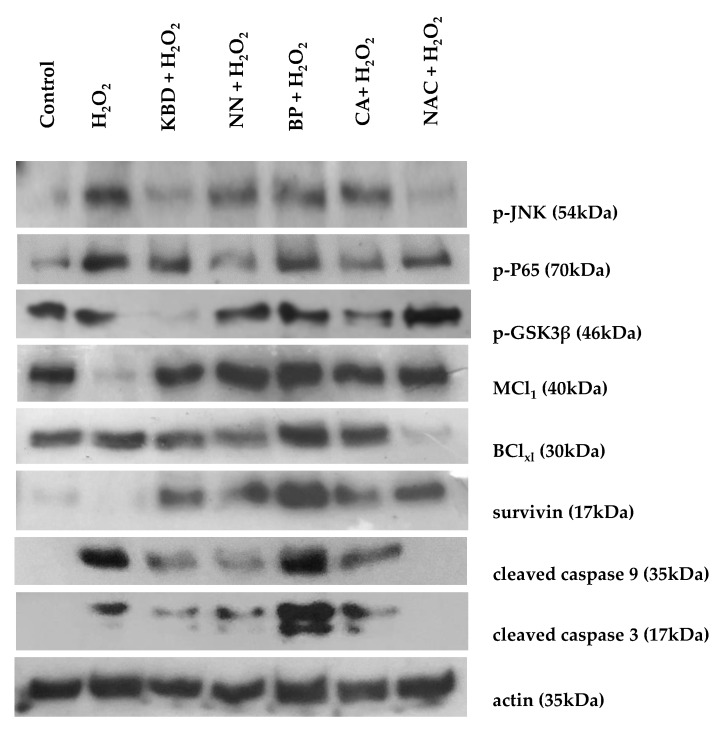
Effect of the KBD (100 μg/mL) and its components (*Nelumbo nucifera* (NN), *Piper nigrum* (BP), and *Centella asiatica* (CA), 35 μg/mL) extracts on protein expression by Western blot analysis. The SH-SY5Y cells were treated with the KBD extract for 30 min by using *N*-acetyl cysteine (NAC) as a positive control, and then treated with 250 µM H_2_O_2_ for 10 min. The cell lysate was prepared and signaling proteins were detected using antibodies against the following: (i) p-JNK, (ii) p-P65, (iii) p-GSK3β, (iv) MCL_1_, (v) BCl_xl_, (vi) survivin, (vii) cleaved caspase-9, (viii) cleaved caspase-3, and (ix) actin.

To identify the protective mechanisms of the ethanol extracts of the KBD and its constituents against H_2_O_2_-induced cell damage, the effect of the extracts on the expression of pro- and anti- apoptotic proteins in the SH-SY5Y neuroblastoma cell line was investigated using Western blot analysis (Figure 5). Treatment with H_2_O_2_ alone increased expression of the pro-apoptotic proteins (cleaved caspase-9, cleaved caspase-3, p-GSK3β, p-P65, and p-JNK) and decreased expression of the anti-apoptotic proteins (MCl_1_ and survivin). In comparison, treatment with KBD extract (100 μg/mL) downregulated expression of the following pro-apoptotic proteins: (i) cleaved caspase-9, (ii) cleaved caspase-3, (iii) p-GSK3β, (iv) p-P65, and (v) p-JNK. Furthermore, treatment with the individual KBD components (NN, BP, and CA) at 35 μg/mL showed some effects on signaling protein expression. The NN extract inhibited JNK and p65 phosphorylation and cleavage of caspase-9 and -3, while increasing levels of MCl_1_ and survivin. The BP extract also inhibited JNK and p65 phosphorylation while increasing MCl_1_, BCL_xl_, and survivin expression but had no effect on caspase-9 and -3 activation. For the CA extract, the cleavage of caspase-9 and -3, as well as phosphorylation of GSK3β, P65, and JNK were all inhibited. However, expression of anti-apoptotic proteins MCl_1_, BCL_xl_, and survivin was increased. Thus, the KBD neuroprotection was due to the combination of reduced expression of pro-apoptotic genes and increased expression of anti-apoptotic genes. 

### 2.7. Behavioral Experiments

#### 2.7.1. Morris Water Maze Test

We examined the effects of the KBD on scopolamine-induced memory impairment in ICR mice using the Morris water maze and the Y-maze tasks. 

**Figure 6:**
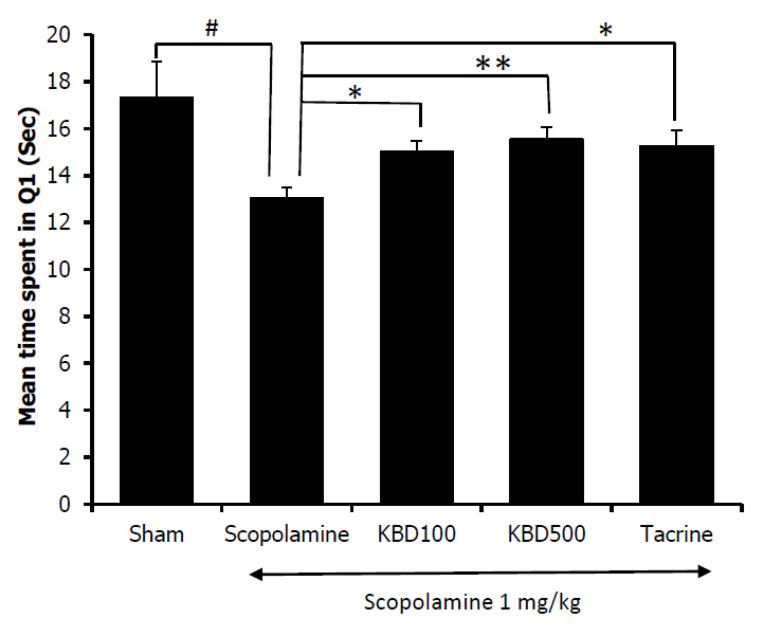
Effect of the KBD (100 and 500 mg/kg) on memory impairment induced by scopolamine in the water maze test. Tacrine at the dose of 10 mg/kg/day was used as a reference standard. The data were shown as mean ± SEM (*n* = 8–10). ^#^ *p* < 0.05 versus control group (Sham), * *p* < 0.05 and ** *p* < 0.01 versus scopolamine-treated group.

Assessment of the effect of the KBD on scopolamine-induced memory impairment was conducted after the mice had been treated with KBD for seven days. The water maze results showed that the mice in the control group spent more time in the target quadrant (Q1) than those in the scopolamine-treated group (Figure 6). The result indicated that the scopolamine-treated mice were in an amnesic state. Tacrine (10 mg/kg), as a positive control, significantly improved cognitive deficits indicated by the mice spending more time in the target quadrant than the amnesic mice. Mice treated with the KBD at the dose of 100 and 500 µg/mL also spent significantly more time in the target quadrant than the scopolamine-treated group. The result indicated that the KBD could improve long-term memory in mice with amnesia induced by scopolamine.

#### 2.7.2. Y-Maze Test

**Figure 7:**
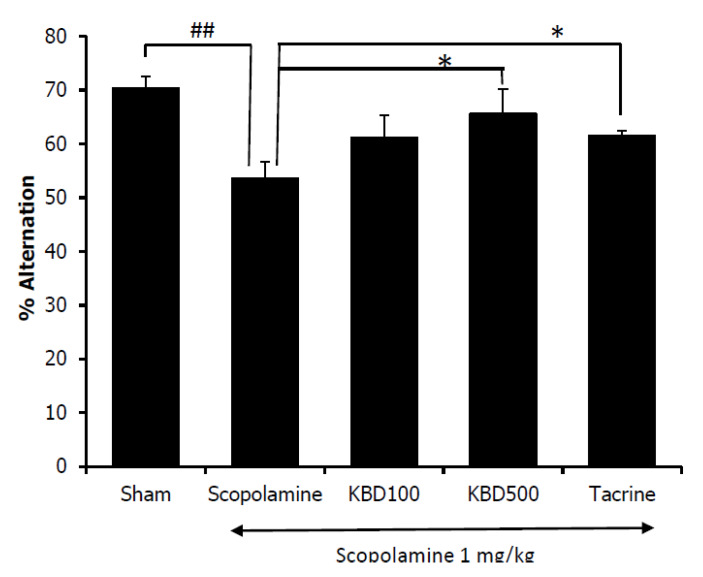
Effect of the KBD (100 and 500 mg/kg) on memory impairment induced by scopolamine in Y-maze test. Tacrine at the dose of 10 mg/kg/day was used as a reference standard. The data were shown as mean ± SEM (*n* = 8–10). ^##^ *p* < 0.01 versus control group (Sham), * *p* < 0.05 versus scopolamine-treated group.

Spontaneous alteration behavior in the Y-maze test is considered to correlate with immediate spatial working memory, a form of short-term memory [16]. The percentage of alternation behavior was measured after seven days of KBD treatment. As shown in Figure 7, the results showed that the mice in the scopolamine-treated group had a reduced alternation percentage as compared with the control group (*p* < 0.01), and the reduced alternation induced by scopolamine was significantly reversed by the KBD at 500 mg/kg/day (*p* < 0.05).

## 3. Discussion

Due to the multifaceted pathogenesis of AD, the agents targeting at multiple stages of the pathogenic cascade seem to constitute a potential drug for AD treatment. Compounds from natural plants, the latter a rich source of chemical diversity, can create a potential multitarget drug for disease treatment. The KBD consists of three herbal plants relevant to AD treatment; thus, the KBD can have good potential for the treatment of AD. Hence, this study was conducted to investigate the potential of the KBD for AD treatment. 

Oxidative stress has been widely associated in the development and progression of neuronal cell death which, in turn, leads to neurodegenerative disorders including cognitive decline in AD. Oxidative stress reflects an imbalance between the generation of free radicals and an individual’s antioxidative defense system [31]. Hence, antioxidant drugs can counteract the neurotoxic and harmful effects of oxidative stress and alleviate the progress of AD. Several studies have shown that treatment with antioxidants could delay the development of AD [32,33]. In the present study, the in vitro antioxidant activity of the KBD and its components correlated with the total phenolic content of crude extracts. This is consistent with a previous study that reported strong correlations between total phenol content and radical scavenging activity [34]. The radical scavenging activity of the KBD is likely to be provided by NN, which has the highest phenolic content. 

It has long been recognized that the cholinergic system plays a vital role in a number of cognitive functions [35]. Evidence has suggested that memory deficit in AD results from a decline of acetylcholine (ACh), which is the neurotransmitter that plays roles in learning and memory functions [36]. Hydrolysis of ACh through AChE results in termination of cholinergic transmission, therefore, inhibition of AChE serves as a therapeutic target for AD treatment. This research has demonstrated that the KBD extract showed an AChE inhibitory effect in a dose-dependent manner. Furthermore, the AChE inhibitory activity of the KBD mainly comes from BP, which has a high flavonoid content. This outcome correlates with a previous study showing that several flavonoids possess AChE inhibitory activity [37]. 

Another key neuropathological hallmark of AD is the accumulation of toxic Aβ plaques in the brain. Therefore, halting the pathological Aβ aggregation is the principal goal of several therapeutic strategies in preclinical development or in clinical trials. Aβ_1-42_ is a main composition of amyloid plaques evident in the AD brain and several studies have indicated that Aβ_1-42_ is an important factor in the etiology of AD [38,39]. In vitro and transgenic mice studies have shown that Aβ_1-42_ aggregates to form amyloid plaques faster than Aβ_1-40_. Therefore, we employed Aβ_1-42_ to examine Aβ aggregation. The KBD extract, NN, BP, and CA all inhibited the aggregation of Aβ_1-42_ in the in vitro assay. In addition, the Aβ aggregation inhibition activity had a good correlation with phenolic content. Thus, it appears the Aβ aggregation inhibitory activity of the KBD extract mainly comes from NN. 

Reactive oxygen species (ROS) possess the ability to damage neuronal cell via apoptosis or necrosis. An exogenous source of ROS, such as H_2_O_2_, is widely used to cause apoptosis (mild oxidative stress) and necrosis (severe oxidative stress) [40]. Several instances revealed etiological associations between the production of H_2_O_2_ and neurodegenerative diseases [41]. Thus, H_2_O_2_-induced neuronal damage is considered to be a suitable model for the study of neurodegeneration induced by oxidative stress [42]. Pretreatment of SH-SY5Y cells with the KBD significantly increased the viability of cells exposed to H_2_O_2_. Of the individual components, only NN showed any improvement in cell viability as compared with the H_2_O_2_ treated control. This neuroprotective action of the KBD can partly come from the antioxidant activity of the extract. However, the neuroprotective ability of its components was not related to antioxidant activity. Our results indicate that BP, which has a higher antioxidant capacity than CA, showed a less neuroprotective effect. Apart from the antioxidant action, the protective effect could come from other mechanisms. Therefore, to identify the protective mechanisms of the ethanol extracts of the KBD and its constituents against H_2_O_2_-induced cell death, we investigated the effect of the KBD and its components on the expression of pro- and anti-apoptotic proteins using Western blot analysis. Pretreatment of SH-SY5Y cells with the KBD extract or its components (NN, BP, and CA) prior to H_2_O_2_ exposure showed downregulation of the following pro-apoptotic proteins: (i) cleaved caspase-9, (ii) cleaved caspase-3, (iii) GSK-3β, (iv) p-P65, and (v) p-JNK and upregulation of the following anti-apoptotic proteins: (i) MCl_1_, (ii) Bcl_xl_, and (iii) survivin. Previous studies have reported that oxidative stress-induced neuronal cell death in H_2_O_2_-induced SH-SY5Y cells is associated with the mitochondrial apoptotic pathway and is an important mechanism in neurodegenerative diseases. The excessive ROS generation causes the increase of mitochondrial membrane permeability, resulting in the release of cytochrome C. Released cytochrome C activates pro-apoptotic factors, such as caspase-9 and -3, and eventually induces cell death. Our results show increased proteolytic cleavage of caspase-9 and -3, and increased phosphorylation of p-GSK-3β, p-P65, and p-JNK following treatment with H_2_O_2_. The KBD protects neuronal cells against H_2_O_2_-induced death by downregulation of cleaved caspase-9 and cleaved caspase-3, decreased phosphorylation of GSK-3β, P65, and JNK, as well as increased expression of MCl_1_, Bcl_xl_, and survivin. Therefore, the KBD appears to protect against neuronal cell death induced by oxidative stress through both its antioxidant and anti-apoptosis actions. 

Taken together, the in vitro results indicate that the KBD possesses multimodes of action against multiple targets in the AD pathological cascade including: (a) antioxidant, (b) anti-AChE, (c) anti-Aβ aggregation, (d) neuroprotection, and (e) anti-apoptosis. These activities resulted from different phytochemical contents and from different plants that comprise the KBD. Due to the multifaceted pathogenesis nature of AD, various classical agents were proved to be ineffective because they were mostly designed to cope with only one mode of action. Thus, the KBD which demonstrated multimode of actions is more likely to possess higher potential for AD treatment. Our data also proved that the KBD revealed additional benefits as compared with the activities of its individual plant in the composition. The KBD demonstrated a broader mode of action against AD as compared with its individual plant formulation. Due to a lower dose of each component, the KBD could generate less toxicity for a long-term treatment of AD.

Furthermore, we examined the effect of the KBD on memory deficit induced by scopolamine in mice. The nonselective muscarinic antagonist scopolamine has frequently been used to impair learning and memory. Scopolamine-induced amnesia is the classical approach to evaluate the effects of novel cognition-enhancing drugs in animals [43].

The Morris water maze was employed to assess the long-term memory of the experimental animals [44]. On testing day, if the mice spent more time in the target quadrant where the platform had previously been located during the training trials, this outcome would indicate spatial memory improvement [35]. In contrast, the Y-maze investigated working memory or short-term memory by observing the animal’s spontaneous alternation behavior. Mice with short-term memory loss could not recall which arm they had just visited, and thus showed reduced spontaneous alternation. Our results showed that the seven-day KBD treatment reversed scopolamine-induced memory deficit. The treated mice demonstrated increased spontaneous alternation in the Y-maze test and increased the time spent in the quadrant where the platform had previously been placed in the water maze test. Our in vivo results indicated that the KBD could improve both short-term and long-term memories in amnesic mice whose memory loss was induced by scopolamine. Several studies have indicated that scopolamine impaired the cholinergic system’s performance by acting on muscarinic receptors and increasing AChE activity. From our in vitro AChE function assay, the KBD demonstrated AChE inhibitory action. Tacrine also works by inhibiting AChE. Both KBD and tacrine showed improvement in memory deficit induced by scopolamine, which suggests that they act via a similar mechanism. Moreover, a previous study reported that NN, one of the components of the KBD, reversed scopolamine induced memory impairment by increasing ChAT expression [23]. Therefore, the cognitive improvement resulting from treatment by the KBD could partly come from the AChE inhibitory action, coupled with stimulation of ChAT expression.

## 4. Materials and Methods 

### 4.1. Chemicals and Reagents 

The KBD and its three components as follows: (i) *Nelumbo nucifera* petals (NN), (ii) *Piper nigrum* fruits (BP), and (iii) the aerial part of *Centella asiatica* (CA) were provided by Chao Phya Abhaibhubejhr Hospital, Prachinburi Province, Thailand. The plants were identified by Benjawan Leenin, a chief of Traditional Knowledge Center, Chao Phya Abhaibhubejhr Hospital Foundation. The relative herbarium voucher specimens were deposited at the museum of Chao Phya Abhaibhubejhr Hospital with following voucher numbers: ABH15, ABH18, and ABH17, respectively. Acetylthiocholine iodide (ATCI), bovine serum albumin (BSA), beta amyloid 1-42 (Aβ_1–42_), gallic acid, quercetin, β carotenoid, tacrine, trolox, *N*-acetyl cysteine (NAC), trypsine, fetal bovine serum (FBS), and Dulbecco's modified Eagle medium nutrient mixture F-12 (DMEM/F12) were purchased from Sigma-Aldrich (SM Chemical supplies Co., Ltd., Bangkok, Thailand), Merck (Merck, Bangkok, Thailand), Gibthai (GT Chemical supplies Co., Ltd., bangkok, Thailand), and Fluka (SM Chemical supplies Co., Ltd. ,Bangkok, Thailand).

### 4.2. Preparation of KBD and Its Components Extracts

Crude extracts of KBD, NN, BP, and CA were prepared by macerating with ethanol at 1:5 (*w*/*v*) ratios for 3 days and repeated twice. Concentrated extracts were obtained by filtration, and then evaporation using a rotary evaporator. The concentrated extracts were lyophilized into powder and stored in air-tight containers at 2–8 °C until needed for use. The yields of the crude extracts were KBD (11.32%), NN (5.26%), BP (6.40%), and CA (5.90%) of dry weight. The HPLC analysis of the KBD extract has been reported in a previous study [20]. Before using, the extract powder was prepared as stock solution in ethanol or DMSO with the concentration of 10 mg/mL.

### 4.3. Assessment of Total Phenolic and Total Flavonoid Contents

The total phenolic content was determined by dissolving KBD, NN, BP, and CA crude extracts in ethanol, and then 10 μL of each extract was combined with 75 μL of Folin–Ciocalteu reagent. The ingredients were mixed and left for 5 min, prior to the addition of 75 μL of 7.5% sodium carbonate solution. After 2 h of incubation, absorption at wavelength 700 nm was measured. The total phenolic content of each extract is shown as μg of gallic acid equivalents per mg of crude extract (μg GAE/mg CE) against a calibration curve with gallic acid. All samples were analyzed in three replicates [45].

The total flavonoid content assay was performed by adding the following: (i) 20 μL of sample, (ii) 15 μL of aluminum chloride solution (2.5%), (iii) 20 μL of sodium acetate (100 g/L), and (iv) 145 μL of distilled water, into 96-well plates. The mixture was left at room temperature for 15 min and absorbance was detected at a wavelength of 450 nm. Quercetin was used as a reference standard. The total flavonoid content is shown as μg of quercetin equivalents per mg of crude extract (μg QE/mg CE) against the calibration curve of quercetin [46].

### 4.4. In Vitro Antioxidant Activities Assays 

The antioxidant activities of ethanol extracts of KBD, NN, BP, and CA were investigated by the following two methods: (i) ABTS (2,2′-azino-bis-3-ethylbenzthiazoline-6-sulphonic acid) and (ii) DPPH (1,1-diphenyl-2-picrylhydrazyl). 

The ABTS^•+^ mixture was prepared by incubating 7 mM ABTS with 2.45 mM potassium persulfate for 16 h in the dark. Ethanol was added to the ABTS^•+^ solution to achieve absorbance of 0.80 ± 0.02 at 700 nm. Then, 50 µL of various concentration of crude extracts in ethanol were mixed with 100 µL of the ABTS^•+^ mixture. The absorbance at 700 nm was determined 15 min after initial mixing [16]. Trolox served as a positive control.

The DPPH assay was carried out by incubating 100 µL of various concentrations of crude extracts in ethanol with 100 µL of 0.2 mM DPPH solution for 30 min. The absorbance was determined at 550 nm using a microplate reader [47]. The ability to scavenge free radicals was calculated using this equation:

Percentage inhibition = (A_C_ − A_S_)/A_C_ × 100 (1)

where A_C_ is absorbance of control and A_S_ is absorbance of the sample.

### 4.5. In Vitro AChE Inhibitory Activity Assay

The AChE inhibitory activities of ethanol extracts of KBD, NN, BP, and CA were investigated by using the modified Ellman’s method [48]. The assay was conducted with microplates in triplicate by mixing the following: (a) 25 µL of 1 mM ATCI, (b) 25 µL of 0.1 M phosphate buffer, (c) 25 µL of the extracts, and (d) 125 µL of 1 mM DTNB with 50 µL of AChE from an electric eel type VI-S (0.2 Units/mL). At least 5 concentrations of extracts were determined. The absorbance at 405 nm was monitored once every 30 s for 5 min. The enzymatic activity and percentage inhibition were determined. The inhibitory activity can be calculated from enzymatic activity by the following equation:

Percentage inhibition = [(A_E_ − A_S_)/(A_E_ − A_C_)] × 100 (2)

where A_E_, A_S_, and A_C_ are the enzymatic activity of enzyme, sample, and control, respectively.

### 4.6. In Vitro Assay for Aβ Aggregation Inhibition 

A thioflavin-T (ThT) fluorescence assay was operated to monitor the aggregation of amyloid β (Aβ1–42) [49]. Curcumin at the concentration of 10 μM was used as a reference standard. Briefly, 25 μM of Aβ1–42 in 50 mM phosphate buffer pH 7.4 was incubated with KBD (300 μg/mL), NN, BP, or CA (100 μg/mL), or curcumin (10 μM) for 48 h at 37 °C. After incubation, the mixtures were combined with 5 μM ThT in glycine/NaOH buffer pH 8.0. Fluorescence intensity was measured with excitation/emission wavelengths of 446/490 nm. The percentage inhibition of aggregation was obtained by using the following equation:

Percentage inhibition = (1 − IFs/IFc) × 100%(3)

where IFs and IFc were the fluorescence intensities of sample and control, respectively.

### 4.7. Effect on Hydrogen Peroxide-Induced Cell Damage in Neuroblastoma Cells

Neuroblastoma cells (SH-SY5Y) were maintained in DMEM/F12 containing 10% FBS at 37 °C in a humidified 5% CO_2_ incubator. Then, cells were plated at a density of 2.5 × 10^5^ cell/mL into a 96-well plate for all experiments. The cells were pretreated with 100 μg/mL KBD, its components NN, BP, or CA at equivalent amounts (35 μg/mL) or standard reference NAC (100 µg) for 2 h, and then incubated with or without hydrogen peroxide (H_2_O_2_) at a concentration of 250 μM for 2 h [15,16]. Cell viability was detected by staining the cells with 3-(4,5-dimethyl-2-thiazolyl)-2,5-diphenyl-2*H*-tetrazolium bromide (MTT) (5 mg/mL in PBS) for 2 h. The absorbance was recorded by a well plate reader at 600 nm. All data were represented as percentages of non-H_2_O_2_-treated groups (control group). The cell viability of control group was expressed as 100%. 

### 4.8. Effect on Protein Expression Induced by H_2_O_2_ in Neuroblastoma Cells

The SH-SY5Y cells were maintained in DMEM/F12 containing 10% FBS at 37 °C in a humidified 5% CO_2_ incubator. Cells at a density of 1 × 10^6^ cell/mL were plated into a 6-well plate. The cells were pretreated with 100 μg/mL KBD, its components NN, BP, or CA at equivalent amounts (35 μg/mL) or the standard reference NAC (100 µg) for 30 min. After washing the unabsorbed substances, the cells were treated with H_2_O_2_ 250 μM for 15 min. Then, to lyse cells, the treated cells were mixed with an ice-cold lysis buffer for 30 min. The lysate was centrifuged at 13,500 rpm for 10 min at 4 °C and the supernatant was taken. The total protein concentration was quantified by using the Bradford assay. Proteins were separated using SDS-PAGE and transferred to a polyvinylidene difluoride (PVDF) membrane. The membrane was treated with BlockAce and probed with primary antibodies (cleaved caspase-9, cleaved caspase-3, p-GSK-3β, p-P65, and p-JNK, as well as the anti-apoptotic proteins MCl_1_, Bcl_xl_, and survivin). The antibodies were monitored using horseradish peroxidase-conjugated anti-rabbit, or anti-goat secondary antibodies and visualized with the enhanced chemiluminescence system [50]. 

### 4.9. The Effects of KBD on Scopolamine-Induced Memory Impairments in Mice 

The study was conducted on male ICR mice weighing between 25 and 35 g, derived from the National Laboratory Animal Centre, Nakorn Pathom, Thailand. All animal studies were approved by the Institutional Animal Care and Use Committee of Khon Kaen University, Thailand (record no. IAC UC-KKU-25/62). The mice were kept in groups of five per cage for seven days in the animal house, at an ambient temperature of 25 °C with humidity level of 50–55% and a 12 h diurnal light cycle, prior to testing. Water and food were freely available in their home cages. 

The effect of KBD on scopolamine-induced memory deficit in mice was investigated using two behavioral models, i.e., water maze and Y-maze tests. After treatment with the KBD powder (100 and 500 mg/kg/day, oral (p.o.) administration) or a reference standard (10 mg/kg/day of tacrine, p.o.) for seven days, male ICR mice were examined for behavioral changes in learning and memory. The KBD dose was calculated from the clinical dose (2000 mg/day), which was prescribed in the hospital [20]. This dose was converted into the appropriate dose for mice, according to the following equation: human equivalent dose (HED, mg/kg) = mouse dose (mg/kg) x (mouse Km/Human Km), where Km is the correction factor [51]. For each experiment, after treatment with test compounds for 1 h, the memory deficit was induced by scopolamine. Thirty minutes after scopolamine administration, all animals were evaluated for memory deficit by Morris water maze and Y-maze tests.

The Morris water maze [44] was composed of a black circular pool (70 cm in diameter and 28 cm high) filled with water (25 ± 1 °C) and a dark removable platform (6 × 10 × 15 cm) placed 1 cm beneath water surface in a targeted quadrant (Q1). For the training session, the mice were introduced into various quadrants of the pool (Q1–Q4) and allowed to swim for 60 s in search of the platform. The time spent to reach the platform was recorded as ”escape latency”. Each animal was subjected to four trials per day for 5 consecutive days. For the test session, the mice were released in the water pool without platform and allowed to swim from the various quadrants for 60 s. The swimming time was recorded in the target quadrant (Q1) where the platform had been placed. 

For the Y-maze test [16], the Y-maze apparatus was made of black polypropylene with three equally spaced arms at a 120° angle from each other. Each arm was 40 cm long, 10 cm wide at the top, 3 cm wide at the bottom, and 12 cm high. Each mouse was positioned at the end of one arm and allowed to move freely through the maze for 8 min. The total number of arm entries and the sequence of entries were manually recorded in order to calculate the percentage of alternation. The alternation behavior was classified by consecutive visits into all three arms without repeated entries. The alternation was represented as the ratio of alternation to possible alternation multiplied by 100.

### 4.10. Statistical Analyses

The results are represented as mean ± SD (*n* = 4–6) and mean ± SEM (*n* = 8–10) for in vitro and in vivo experiments, respectively. Statistical significance was determined by student t-test or one-way analysis of variance (ANOVA). For all statistical analysis, significance levels were set at a *p* value < 0.05.

## 5. Conclusions

The results from the present study indicate that the KBD possesses multiple modes of action aiming at multiple targets in the AD pathology cascade which include: (i) AChE inhibition, (ii) free radical scavenging action, (iii) anti-amyloid β aggregation, and (iv) neuroprotection against H_2_O_2_ induced damage. The KBD reduced H_2_O_2_-induced neuronal cell death by inhibiting pro-apoptotic factors, specifically the following: (a) blocking caspase-9 and (b) caspase-3 cleavage activation, (c) changing phosphorylation levels of P65, (d) JNK, and (e) GSK-3β, while activating the following anti-apoptotic factors: (a) MCl_1_, (b) BCl_xl_, and (c) survivin. Furthermore, the KBD exhibited an ability to improve scopolamine induced memory deficit in mice. Our results illustrate that the Kleeb Bua Daeng formula has the potential to be employed in AD treatment. Thus, the Kleeb Bua Daeng formula could be used as an alternative novel choice for the prevention and treatment of patients with Alzheimer’s disease.

## Figures and Tables

**Table 1:** Total phenolic and flavonoid contents in the Kleeb Bua Daeng formula (KBD) and its components. The total phenolic and flavonoid contents of each extract are shown as μg of gallic acid equivalents per mg of extract (μg GAE/mg Extract) and as μg of quercetin equivalents per mg of extract (μg QE/mg Extract), respectively. Data are represented as mean ± SD (*n* = 3). Different letters in the same column are significantly different (*p* < 0.05).

Samples	Total Polyphenols (μg GAE/mg Extract)	Total Flavonoids (μg QE/mg Extract)
KBD	22.57 ± 0.63 ^a^	31.17 ± 1.48 ^a^
*Nelumbo nucifera* (NN)	43.85 ± 0.46 ^b^	33.83 ± 2.22 ^a^
*Pipper nigrum* (BP)	29.98 ± 0.63 ^c^	42.12 ± 5.54 ^b^
*Centella asiatica* (CA)	12.64 ± 0.25 ^d^	19.21 ± 0.59 ^c^

**Table 2:** In vitro ABTS and DPPH radical scavenging and acetylcholinesterase (AChE) inhibitory actions of the ethanol extracts of the KBD and its components. Data are represented as mean ± SD (*n* = 3). Different letters in the same column are significantly different (*p* < 0.05).

Samples	ABTS Assay ^a^	DPPH Assay *	AChE Assay **
	IC_50_ (mg/mL)	IC_50_ (mg/mL)	IC_50_ (mg/mL)
KBD	0.90 ± 0.06 ^a^	0.62 ± 0.06 ^a^	2.17 ± 0.23 ^a^
*Nelumbo nucifera* (NN)	0.56 ± 0.03 ^a^	0.26 ± 0.00 ^a,b,^	1.88 ± 0.10 ^a^
*Pipper nigrum* (BP)	0.72 ± 0.02 ^a^	0.73 ± 0.07 ^a,c^	0.93 ± 0.12 ^a^
*Centella asiatica* (CA)	1.91 ± 0.06 ^a^	0.96 ± 0.02 ^a,c^	>5 ^b^
Trolox (μM)	73.14 ± 2.17	22.91 ± 0.16	-
Tacrine (µM)	-	-	0.29 ± 0.03

* Data are expressed as IC_50_, the crude extract concentration that inhibits 50% of free radicals (mean ± SD). ** Data are expressed as IC_50_, the crude extract concentration that inhibits 50% of AChE activity (mean ± SD).
